# Transient receptor potential canonical (TRPC) channels in diabetes and associated complications

**DOI:** 10.1080/19336950.2026.2672357

**Published:** 2026-05-11

**Authors:** Yi Wu, Shengming Chen, Xiaolu Xia

**Affiliations:** a Collaborative Innovation Center for Biomedicine, Shanghai University of Medicine and Health Sciences, Shanghai, China; b School of Pharmacy, Shanghai University of Medicine and Health Sciences, Shanghai, China

**Keywords:** TRPC channels, insulin secretion, insulin resistance, diabetes, diabetic complications

## Abstract

TRPC channels are widely expressed in various tissues and cell types, and accumulating evidence indicates that they play critical roles in pancreatic β cell function, including the regulation of insulin secretion. Moreover, TRPC channels have been implicated in the pathogenesis of type 2 diabetes mellitus (T2DM) and its associated complications, underscoring their potential as therapeutic targets. Despite this, there remains a lack of comprehensive, up-to-date reviews summarizing the distribution and functional roles of TRPC channels in diabetes and its complications. In addition, the expression patterns and physiological significance of certain TRPC subtypes remain controversial. This review, therefore, aims to provide a thorough overview of current research, starting with general aspects of TRPC channel structure and function, and progressing to their physiological and pathological roles, with particular emphasis on their involvement in insulin secretion, insulin resistance, diabetes, and diabetic complications.

## Introduction

Diabetes mellitus is the most prevalent metabolic disorder in humans, characterized by chronically elevated blood glucose levels. Recent projections estimate that the total number of people living with diabetes will rise to 853 million by 2050 [1]. The pathogenesis of diabetes mellitus involves insufficient insulin secretion, impaired insulin responsiveness, excessive hepatic glucose production, and abnormalities in lipid and protein metabolism. Type 2 diabetes is the most common form of diabetes worldwide, and the causes of type 2 diabetes mellitus (T2DM) are multifactorial and complex [2]. T2DM is widely recognized to result from the interplay between peripheral insulin resistance and a genetically driven susceptibility to β cell dysfunction. The management of T2DM currently relies on pharmacological interventions combined with lifestyle modifications. Despite these approaches, a definitive cure remains unavailable. Consequently, there is an urgent need to develop improved and more effective therapeutic strategies for the treatment of T2DM.

Transient Receptor Potential (TRP) channels were named after a spontaneous mutation identified by Cosens and Manning in a *Drosophila* mutant [3]. In this mutant, the electroretinogram of the compound eye displayed a transient receptor potential rather than the sustained depolarization observed in the wild type under prolonged bright light stimulation [3]. TRP channels are classified into seven subfamilies by amino acid homology: TRPA (Ankyrin), TRPC (Canonical), TRPM (Melastatin), TRPML (Mucolipin), TRPN (NO-mechano-potential, NOMP), TRPP (Polycystin), and TRPV (Vanilloid) [4]. TRPC (Transient Receptor Potential Canonical) channels, the first identified members of the TRP family, are present in a wide variety of tissues [5]. Based on amino acid similarities, the mammalian TRPCs fall into four subsets: TRPC1, TRPC2, TRPC3/6/7, and TRPC4/5 [6]. Among them, TRPC2 has degenerated into a pseudogene in humans [7]. TRPC channels transduce extracellular signals, often via GPCR agonists, into Ca^2 +^ and Na^+^ influx, driving membrane depolarization. Intracellular Ca^2 +^, a universal second messenger, then regulates key processes such as synaptic transmission, insulin secretion, cell growth, and muscle contraction [8]. Consequently, TRPC channels might play important roles in multiple aspects, including Ca^2 +^ homeostasis, membrane depolarization, and insulin exocytosis. Emerging evidence further implicates TRPC channels in metabolism-related disorders, particularly those involving insulin signaling, adipose and vascular function, and β cell physiology [4,9].

TRPC channels are critical in pancreatic β cells, and their dysregulation contributes to β cell dysfunction, insulin resistance, and diabetic complications [10–12]. These findings suggest that TRPC channels may contribute to the pathogenesis of diabetes and represent promising therapeutic targets. Beyond β cell dysfunction, diabetes is commonly accompanied by complications such as neuropathy, retinopathy, vasculopathy, and nephropathy. Notably, TRP channels have also been implicated in these diabetes-associated complications, highlighting their broader therapeutic potential in managing both pancreatic dysfunction and systemic sequelae of the disease.

## TRPC channels

Unlike other TRP subfamilies that were discovered through functional screening or genetic association with disease, mammalian TRPC members were identified based on their sequence homology with the *Drosophila* prototype TRP and TRP-Like (TRPL) proteins [13]. TRPC channels are widely expressed in the cardiovascular and nervous systems and other cell types and tissues, including the lung, placenta, retinal endothelial, kidney, skeletal muscle, adipose tissue, liver, and pancreas [13,14].

### TRPC channels structure

**Figure 1.**
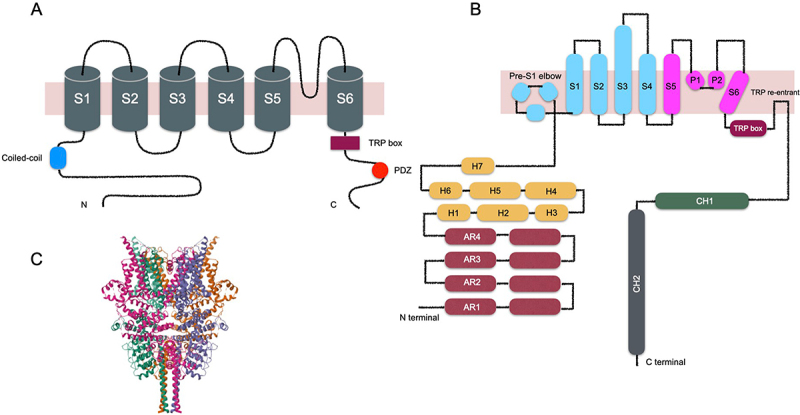
Structures of TRPC channels.

TRPC channels assemble as homo- or hetero-tetramers, with each subunit consisting of six transmembrane segments (S1–S6) and a pore loop between S5 and S6 that forms the ion selectivity filter and pore region [4,15,16]. A three-helix region, the pre-S1 elbow and pre-S1 helix, precedes the S1 helix [17,18]. The cytosolic N- and C-termini co-assemble into a square, dome-like base structure [4,16]. The N-terminal domain contains several ankyrin repeat domains (AR) that mediate protein – protein interactions and facilitate channel assembly, followed by a series of α-helical segments (H1–H7). The C-terminal domain contains a unique common motif, the TRP box (EWKFAR) [19], which is crucial for channel gating [15,20]. This motif is succeeded by a relatively long connecting helix linked to a coiled-coil domain. The connecting helices and coiled-coil domains together mediate ligand binding and channel assembly within the lipid bilayer [21,22]. Between the TRP box and the connecting helix lies a TRP reentrant helix, which is partially embedded in the membrane and, in some structures, presents as a short, open loop rather than a full helix [23,24]. The C-terminal domain also harbors a calmodulin- and IP_3_-receptor – binding (CIRB) region, which is important for Ca^2 +^ /calmodulin regulation [25,26], as well as a PDZ-binding motif that participates in protein – protein interactions [27] (Figure 1).

A, Schematic representation of a single TRPC channel subunit, illustrating the six transmembrane segments (S1–S6), the TPR box, and the cytoplasmic N- and C-terminal domains. B, Topology of a representative TRPC6 subunit, highlighting key structural elements, including the pre-S1 region, ankyrin repeat domains (ARs), and the connecting helix (CH1) and coiled-coil domain (CH2). C, Cryo-EM structure of human TRPC6 at 3.8A resolution. Image from the RCSB PDB (RCSB.org) of PDB ID 5Y×9 (Q. Tang, W. Guo, L. Zheng, J.X. Wu, M. Liu, X. Zhou, X. Zhang, L. Chen, Structure of the receptor-activated human TRPC6 and TRPC3 ion channels (2018) Cell Res. China 28(7): 746–755).

### Activation mechanisms of TRPC channels

TRPC channels are nonselective cation channels that mediate calcium (Ca^2 +^) and sodium (Na^+^) influx in response to various physiological signals. Their Ca^2 +^ /Na^+^ selectivity ratio varies among subtypes [28]. Activation typically occurs via G protein – coupled receptors (GPCRs) or tyrosine kinase receptors mediated activation of phospholipase C (PLC). PLC catalyzes the hydrolysis of phosphatidylinositol 4,5-bisphosphate (PIP_2_) in the plasma membrane, generating diacylglycerol (DAG) and inositol 1,4,5-trisphosphate (IP_3_) [13,29,30]. IP_3_ binds to IP_3_ receptors on the endoplasmic reticulum (ER), triggering Ca^2 +^ release from the ER lumen and leading to intracellular Ca^2 +^ store depletion [31]. TRPC channels can also be activated by lysophospholipids, hypoosmotic stress, and mechanical stimuli [14]. Because the PLC cascade comprises multiple regulatory steps, several components can influence TRPC gating. Activation of TRPC1/4/5 homo- or heterotetramers typically depends on IP_3_ binding to ER IP_3_ receptors, which in turn triggers extracellular Ca^2 +^ entry via store-operated calcium entry (SOCE) [32–35]. TRPC3, TRPC6, and TRPC7 are directly activated by DAG, a PIP_2_ degradation product [13,29,36], a mechanism referred to as receptor-operated calcium entry (ROCE) [34,35]. Notably, TRPC4 and TRPC5 May also gain DAG sensitivity when Na^+^ /H^+^ exchanger regulatory factor (NHERF) proteins dissociate from their C-termini. PKC-mediated phosphorylation of a threonine in the PDZ-binding motif disrupts NHERF interaction, unveiling a dynamic regulatory mechanism that confers DAG sensitivity [37]. Therefore, it is considered that all the TRPC subtypes could be activated by DAG.

**Figure 2.**
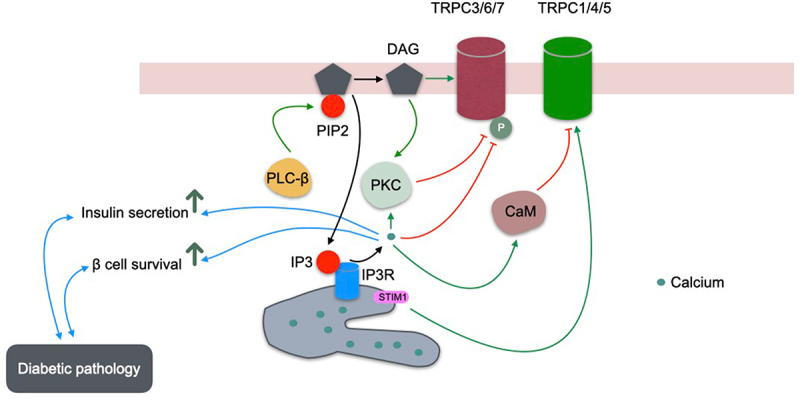
Mechanisms regulating TRPC channel activity.

Several other components in the PLC pathway have also been implicated in TRPC regulation. IP_3_ can enhance TRPC7 activation [38] and modulate TRPC3 and TRPC5 via IP_3_ receptors [39,40]. Indeed, IP_3_ receptors physically associate with TRPC channels [41,42], and direct binding occurs between the IP_3_ receptor N-terminus and the C-terminal CIRB motif of TRPCs across all receptor isoforms and TRPC subtypes [25,26]. TRPC3, TRPC4, and TRPC1-containing channels are activated by IP_3_ receptors but inhibited by Ca^2 +^ –calmodulin, which competes with IP_3_ receptors for the CIRB motif in a mutually exclusive manner [25,26,40,43]. Calcium also modulates TRPC channel activity, enhancing TRPC5 while inhibiting TRPC3 and TRPC6, often via calmodulin or other Ca^2 +^ -binding proteins [44]. PIP_2_ is crucial for sustaining TRPC activity [45–47], though its depletion effects are debated. Förster resonance energy transfer (FRET) analyses indicate that TRPC activation correlates with PIP_2_ hydrolysis, whereas channel inactivation coincides with PIP_2_ dissociation [48] (Figure 2).

Receptor activation triggers phospholipase C (PLC), which hydrolyzes phosphatidylinositol 4,5-bisphosphate (PIP_2_) to generate inositol 1,4,5-trisphosphate (IP_3_) and diacylglycerol (DAG). IP_3_ then binds to ER IP_3_ receptors, releasing stored Ca^2 +^ and depleting ER Ca^2 +^ stores. DAG can directly activate TRPC3, TRPC6, and TRPC7 channels at the plasma membrane. Additionally, DAG, together with Ca^2 +^, can activate protein kinase C (PKC), which phosphorylates TRPC channels to inhibit TRPC3/TRPC6 activity. Additionally, Ca^2+^ may regulate TRPC channels in both calmodulin (CaM)-dependent and -independent manners. TRPC1, TRPC4, and TRPC5 can also be directly activated by stromal interaction molecule 1 (STIM1), which senses ER Ca^2 +^ store depletion, oligomerizes, and translocates toward the plasma membrane to interact with TRPC channels. Calcium is the center of this TRPC network and regulates both insulin release and cell survival, which are linked to diabetic pathology.

### Subtypes of TRPC channels

#### TRPC1

TRPC1, the first mammalian TRP channel cloned, is broadly expressed across multiple tissues [49,50]. It likely serves as an auxiliary subunit in heteromeric TRPC channels [51–53], or even more distantly related channels such as TRPP2 [54], TRPV4 [55], and TRPV6 [56] rather than forming homomeric channels, which remains controversial [20]. In heteromeric channels, it imparts particular gating/selectivity changes [53]. It commonly forms heterotetramers with TRPC4 or with both TRPC4 and TRPC5, particularly in neuronal tissues [57–59], with recent cryo-EM studies revealing a TRPC1:TRPC4 stoichiometry of 1:3 [60]. TRPC1 participates in fundamental cellular processes such as proliferation, differentiation, secretion, migration, and survival [61]. Furthermore, substantial evidence supports that TRPC1 either constitutes or contributes to store-operated channels (SOCs) [62].

#### TRPC2

TRPC2, considered a pseudogene in humans [7], is functionally expressed in mice, rats, and several other mammalian species. In rodents, TRPC2 is highly expressed in the vomeronasal organ [63]. It plays critical roles in olfactory signal transduction and in the regulation of thyroid function [4], as well as in sex discrimination and male – male aggression [64,65]. More recently, studies in mice have shown that TRPC2 contributes to sexual differentiation of the posterodorsal aspect of the medial amygdala and the ventrolateral aspect of the ventromedial hypothalamic nucleus [66].

#### TRPC3

TRPC3 is ubiquitously expressed, although its levels in peripheral tissues are relatively lower than those in the brain [7]. TRPC3 is closely related to TRPC6 and TRPC7, and all three can form homotetrameric or heterotetrameric channels in both native and heterologous systems [67]. TRPC3 adopts a lipid-occupied closed state at 3.3 Å, with four elbow-like helices preceding the first transmembrane segment. Its unusually long S3 helix forms a distinct transmembrane domain that contributes to an extracellular region potentially acting as a sensor for external stimuli [36]. The transmembrane region also contains a DAG-sensing structure, where DAG binding to lipid-interaction sites stabilizes a sensitized closed state, enabling rapid and efficient channel opening upon activation [36,51,68]. TRPC3 functions as a versatile cellular sensor with wide-ranging physiological and pathological significance. It plays a central role in calcium signaling, regulating key processes such as immune responses and vascular tone.

#### TRPC4

TRPC4 mRNA is highly expressed in the brain [69] and bone [70], yet its well-characterized functions extend beyond these tissues, reflecting its broad distribution and functional diversity [71,72]. TRPC4 can assemble into homo- or heterotetramers, frequently pairing with TRPC1 or TRPC5. The resolved cytoplasmic domain of TRPC4 spans ~80 Å, relatively shorter than in other TRPC channels. It exhibits domain swapping of the S5–S6 helices between adjacent subunits, creating a central pore with a negatively charged extracellular opening. A conserved E555 residue forms a stabilizing salt bridge with R556 of the neighboring protomer, a feature shared with TRPC1 and TRPC5 [73]. TRPC4 is involved in numerous physiological and pathological processes, including regulation of vascular tone [74], neuronal excitability [75], angiogenesis [4], smooth muscle contraction [76], and promotion of migration and proliferation of cancer cells [4].

#### TPRC5

TRPC5 is predominantly expressed in the brain, with lower levels in the liver, kidney, testis, and pancreas [77]. TRPC5 is one of the best structurally characterized TRPC channels. TRPC5 shares ~60% sequence identity with TRPC4, and the two exhibit similar overall architectures but differ in extracellular and lipid-interacting domains [78]. A hallmark of TRPC4/5 is a disulfide bridge within the E3 extracellular loop, between C553 and C558 in mTRPC5 or C549 and C554 in mTRPC4, essential for tetramer stability, trafficking, and gating [23,73,78,79]. TRPC5 activity is regulated by cell surface receptors, calcium stores, redox status, and intracellular/extracellular Ca^2 +^ [77]. It displays a bell-shaped response to intracellular Ca^2 +^ [80,81] and is strongly activated by extracellular Ca^2 +^ concentrations above 5 mM [81,82]. TRPC5 is also temperature-sensitive, being potentiated by cooling below 37°C [83]. Channel activation leads to membrane depolarization and Ca^2 +^ influx, influencing angiogenesis, blood pressure control, and endothelium-dependent contraction [4].

#### TRPC6

TRPC6 is abundantly expressed in the placenta, heart, lung, pancreas, kidney, and multiple brain regions [70]. Cryo-EM analysis of the murine TRPC6 cytoplasmic domain at 3.8 Å resolution revealed an inverted dome-like structure with four radial helices converging into a central coiled-coil and a unique domain swap at the helix – coiled-coil junction [15]. TRPC6 contributes to neuroprotection, immune regulation, cardiovascular function, and the development of glomerular injury [4]. It is often studied alongside TRPC3 due to their structural and functional similarities [84–86]; however, their physiological effects can diverge or even oppose one another. For instance, enhanced TRPC3 or diminished TRPC6 activity has been implicated in epilepsy pathogenesis [87].

#### TRPC7

In mice, TRPC7 mRNA is highly expressed in the heart, lung, and eye, with lower expression observed in the brain, spleen, and testis [88]. In humans, TRPC7 mRNA is predominantly expressed in the kidney, pituitary gland, and various brain regions [70]. In terms of its voltage – current characteristics, TRPC7 closely resembles TRPC3 and TRPC6 and can be directly gated by DAG [89,90]. TRPC7 is involved in the initiation of seizures, regulation of electrophysiological functions, aging-associated tumorigenesis, and basal gut smooth muscle excitability [14,91–93].

## TRPC channels on β cells

### Distribution of TRPC channels on β cells

The main TRPC subtypes expressed in β cells are TRPC1, TRPC3, TRPC4, and TRPC6. TRPC1 has been detected in insulinoma cell lines (MIN6, INS-1), mouse and rat islets, rat β cells, and the whole human pancreas [70,94–96]. These isoforms are differentially expressed in β cells due to the physiological roles they play; ones involved in insulin release and cell survival are more abundant [95,97]. TRPC3 is present in rat and mouse pancreatic islets and β cells, and possibly in the human pancreas [97–99]. They are present at very low levels [97,100]. However, some RNA-seq studies have questioned TRPC3 expression in human β cells, likely due to its extremely low abundance [101,102]. TRPC3 is also expressed in pancreatic exocrine acini, where it mediates Ca^2 +^ influx contributing to the pathogenesis of acute pancreatitis [103]. TRPC4 is detected in βTC3, INS-1, MIN6, mouse β cells, and rat β cells [70,104,105]. TRPC6 is identified in MIN6, INS-1, and rat islets [94,106], and although it can be detected in the human pancreas, its presence remains controversial [70,98]. Low levels of TRPC6 are also observed in βTC-3 insulin-secreting cells [105]. Despite these reports, the functional roles of TRPC channels in β cells remain largely uncharacterized, and some literature even questions their existence in these cells, but TRPC may be functionally expressed in human and mouse β cells [97].

### Role of TRPC channels in insulin secretion and insulin resistance

Given that the primary function of TRPC channels is to act as store-operated or receptor-operated channels, replenishing Ca^2 +^ following ER depletion or activation by hormones and neurotransmitters, it is reasonable to speculate that TRPC channels in pancreatic β cells contribute to the regulation of calcium homeostasis, membrane excitability, and cell growth, thereby influencing insulin secretion and β cell function. TRPC channels have been proposed to mediate the depolarizing cation current previously observed in βTC-3 cells [105]. Furthermore, both TRPC3 and TRPC6 have been shown to participate in Pdx-1–activated α- and β cell proliferation [107]. Despite being viewed as functionally distinct, the endocrine and exocrine pancreas exhibit crosstalk that regulates development and function in health and disease [108]. Inhibition of acinar-derived pancreatic elastase promotes β cell proliferation [109]. and because TRPC3 deletion alters Ca^2 +^ signaling and agonist-evoked acinar secretion [103,110], it may disrupt acinar/ductal function and secondarily impair endocrine islets.

#### Insulin secretion

The pancreatic islet consists of glucagon-producing α-cells, insulin-producing β cells, somatostatin-producing δ-cells, pancreatic polypeptide-producing γ (PP)cells, and ghrelin-producing ε-cells [111,112]. Among these, insulin is the only hormone that lowers plasma glucose levels. It is secreted by β cells in response to glucose. Additional secretagogues include amino acids, fatty acids, incretin hormones – such as glucagon-like peptide-1 and glucose-dependent insulinotropic polypeptide – and neurotransmitters, including acetylcholine (ACh) [94]. When glucose levels rise, glucose enters β cells through GLUT1 (human) or GLUT2 (rodent) transporters, resulting in an increase in intracellular ATP levels. Elevated ATP closes ATP-sensitive K^+^ (K_ATP_) channels, leading to membrane depolarization. This process is supported by an unidentified depolarizing background conductance, for which TRP channels have been proposed as potential contributors [106,113]. The resulting depolarization opens voltage-dependent Ca^2+^ channels (VDCCs), and the subsequent increase in intracellular Ca^2+^ triggers insulin vesicle exocytosis [114]. Although glucose-stimulated insulin secretion (GSIS) primarily depends on Ca^2+^ influx through VDCCs, intracellular Ca^2+^ release from the ER via IP3 [115,116] and ryanodine receptors [116,117] also contributes to [Ca^2+^]i regulation and insulin secretion. In mouse β cells, ACh-induced IP3 production depletes ER Ca^2+^ stores, activating SOCs [95,118,119], which further amplify GSIS [116].

Increasing evidence suggests that TRPC channels play an important role in regulating insulin secretion from pancreatic β cells. In INS-1E cells, protein kinase C-α (PKCα) potentiates glucose-stimulated insulin secretion via TRPC1 phosphorylation [120]. Conversely, TRPC1 knockout mice fed a high-fat diet exhibit lower fasting plasma glucose levels compared to wild-type controls [121]. A key function of TRPC1 is its interaction with Orai1 and stromal interaction molecule 1 (STIM1), core components of SOCE. This TRPC1–Orai1–STIM1 complex mediates ACh- and thapsigargin (Tg), a sarco/endoplasmic reticulum Ca^2 +^ -ATPase (SERCA) inhibitor,-induced Ca^2+^ influx and also contributes to GSIS. Inhibition of TRPC1 disrupts GSIS and abolishes the potentiating effects of ACh and Tg. Prolonged exposure to high glucose impairs SOC function, highlighting SOCs as potential therapeutic targets for restoring β cell function in type 2 diabetes [95,122].

Otherwise, TRPC3 has likewise been implicated in GSIS regulation. Activation of G protein-coupled receptor 40 (GPR40) by medium- or long-chain fatty acids amplifies GSIS through TRPC3-mediated Ca^2+^ influx. Blocking TRPC3 inhibits fasiglifam (a GPR40 agonist)-induced increases in [Ca^2+^]i and insulin secretion [99]. Rached et al. further demonstrated that TRPC3-mediated insulin secretion occurs independently of K_ATP_ channels and involves DAG-regulated Ca^2+^ oscillations following glucose stimulation. TRPC3 inhibition or knockout in mice leads to defective insulin secretion and glucose intolerance, while channel activation enhances insulin release and alleviates diabetic symptoms [97]. In contrast, Sabourin et al. reported no effect of TRPC3 blockade on basal or stimulated insulin secretion in INS-1E cells [95], a discrepancy possibly arising from differences in pharmacological tools or cell model systems used.

In βTC-3 cells, Ca^2 +^ depletion activates TRPC4, generating a cationic current that enhances membrane oscillations, Ca^2 +^ influx, and insulin secretion [105,106]. Under fasting conditions, pancreatic β cells exhibit increased surface expression of K_ATP_ channels. Leptin, an adipocyte-derived hormone regulating energy balance and glucose homeostasis, promotes K_ATP_ channel trafficking to the plasma membrane via AMPK activation, which depends on TRPC4 and CaMKKβ, suggesting a role for TRPC4 in β cell excitability and insulin secretion [123]. Similarly, protein histidine phosphatase 1 (PHPT-1), a conserved 14-kDa phosphatase, phosphorylates TRPC4 to promote K_ATP_ channel translocation and insulin secretion [124]. PHPT-1 knockout mice display neonatal hyperinsulinemic hypoglycemia resembling congenital hyperinsulinism (CHI) due to impaired K_ATP_ channel trafficking and reduced TRPC4-mediated Ca^2+^ influx and AMPK activation. These findings highlight PHPT-1 and TRPC4 as essential for normal β cell function and potential contributors to CHI pathogenesis [124]. However, glucose tolerance tests revealed no significant differences in glucose homeostasis between wild-type and TRPC4-deficient mice [125]. Given that TRPC4 is activated through the phospholipase C pathway, it may contribute to the ACh- or glucagon-mediated potentiation of insulin secretion. TRPC4’s involvement in M2/M3 muscarinic receptor signaling further supports its potential role in ACh-mediated insulin secretion [126].

In contrast, TRPC6 inhibition has no effect on blood glucose or plasma insulin during intraperitoneal glucose tolerance tests [97]. Although current evidence supports a role for TRPC channels in insulin secretion, further studies are needed to provide more consistent and comprehensive data to reach definitive conclusions.

#### Glucose uptake and insulin resistance

TRPC channels participate in insulin signaling and glucose uptake in skeletal muscle and adipocytes, and their dysregulation may contribute to insulin resistance. Several TRPC isoforms, like TRPC1, are expressed in metabolically active organs and play roles in cellular energy regulation. Altered TRPC1 function has been linked to insulin resistance and diabetes development. Notably, endothelial TRPC1 deficiency exacerbates high-fat-diet – induced insulin resistance, whereas its overexpression reverses metabolic dysfunction in mouse models [127], consistent with evidence that endothelial dysfunction drives systemic insulin resistance [128].

AKT phosphorylation is a key step in insulin signaling that promotes glucose uptake in target tissues [129]. TRPC3 knockout mice display increased hepatic levels of both phosphorylated and total AKT relative to wild-type controls. Conversely, DAG-mediated TRPC3 activation promotes insulin-dependent glucose transport in skeletal muscle, where TRPC3 colocalizes with GLUT4 [130].

In podocytes, TRPC6 is required for insulin-dependent AMPKα2 activation and glucose uptake. Insulin increases TRPC6–AMPKα2 colocalization, placing TRPC6 upstream of Rho/Rac1 signaling and actin cytoskeleton remodeling necessary for glucose transporter translocation. Inhibition of TRPC6 blocks insulin-stimulated glucose uptake [131]. Moreover, TRPC6 knockout mice display aggravated insulin resistance despite comparable hyperglycemia and blood pressure, accompanied by reduced IRS2 expression and insulin responsiveness in cultured podocytes [132]. Overall, these findings highlight the involvement of TRPC channels in the development of insulin resistance, while emphasizing the need for further investigation to define the contributions of other TRPC family members and to clarify the underlying molecular mechanisms.

## Pathophysiological roles of TRPC channels in diabetes and associated complications

### TPRC channels on diabetes

Though TRPC channels are implicated in regulating glucose-stimulated insulin secretion and glucose uptake, it is not fully understood whether the channels contribute to the pathogenesis of diabetes. Overactivation of TRPCs (especially TRPC3/6) under hyperglycemic or oxidative conditions can cause Ca^2 +^ overload, mitochondrial stress, and β cell apoptosis, whereas reduced TRPC activity impairs insulin exocytosis due to insufficient Ca^2 +^ influx. Pharmacological modulation of TRPCs has been proposed as a therapeutic approach: TRPC inhibitors may protect against β cell death and vascular injury. TRPC activators might enhance insulin secretion in early diabetes stages.

Recent studies have linked polymorphisms in the TRPC1 gene to the development of T2DM [133]. Nonetheless, the specific role of TRPC1 in regulating insulin secretion remains to be elucidated. TRPC1 knockout mice exhibit increased body weight, fasting hyperglycemia, impaired glucose tolerance, and elevated plasma triglycerides and cholesterol compared with wild-type controls. Their livers are also heavier and enriched in triglycerides, suggesting that TRPC1 deficiency disrupts metabolic homeostasis [134].

Activation of TRPC3 by small molecule agonist GSK1702934A alleviates several hallmarks of T2DM in mice, including reductions in fasting blood glucose and HbA1c levels, increased plasma insulin, improved glucose tolerance, and decreased liver weight and lipid accumulation [97]. Conversely, TRPC3 inhibition has been shown to improve diabetic wound healing – a major complication of diabetes characterized by impaired fibroblast function. In diabetic dermal fibroblasts, elevated TRPC3 expression suppresses TGF-β signaling, hindering the fibroblast-to-myofibroblast transition essential for tissue repair. Pharmacological inhibition of TRPC3 restores fibroblast function via TGF-β1/SMAD4 upregulation, identifying TRPC3 as a therapeutic target for diabetic wound healing [135].

T2DM is associated with heightened sympathetic activity. In diabetic rats, TRPC1/4/5/6 expression and TRPC currents are increased in the paraventricular nucleus (PVN) and arcuate nucleus (ARCN), and leptin microinjection into these nuclei augments renal sympathetic nerve activity and blood pressure. These effects are abolished by TRPC inhibition, indicating that TRPC activation mediates leptin-induced sympathoexcitation in T2DM [136]. Collectively, all these findings support a central role for TRPC channels in the pathogenesis of diabetes.

Notably, TRPC channels have also been implicated in type 1 diabetes. TRPC6 knockout in type 1 diabetic mice worsens insulin resistance, and global TRPC6 deletion exacerbates glomerular injury in Akita mouse models of type 1 diabetes [132].

In summary, TRPCs are primarily involved in the physiological regulation of blood glucose in early diabetes and pre-diabetic states. However, in the late stage of diabetes, the persistent metabolic stress dysregulates TRPCs, contributing to diabetic complications.

### TPRC channels on diabetic complications

**Figure 3.**
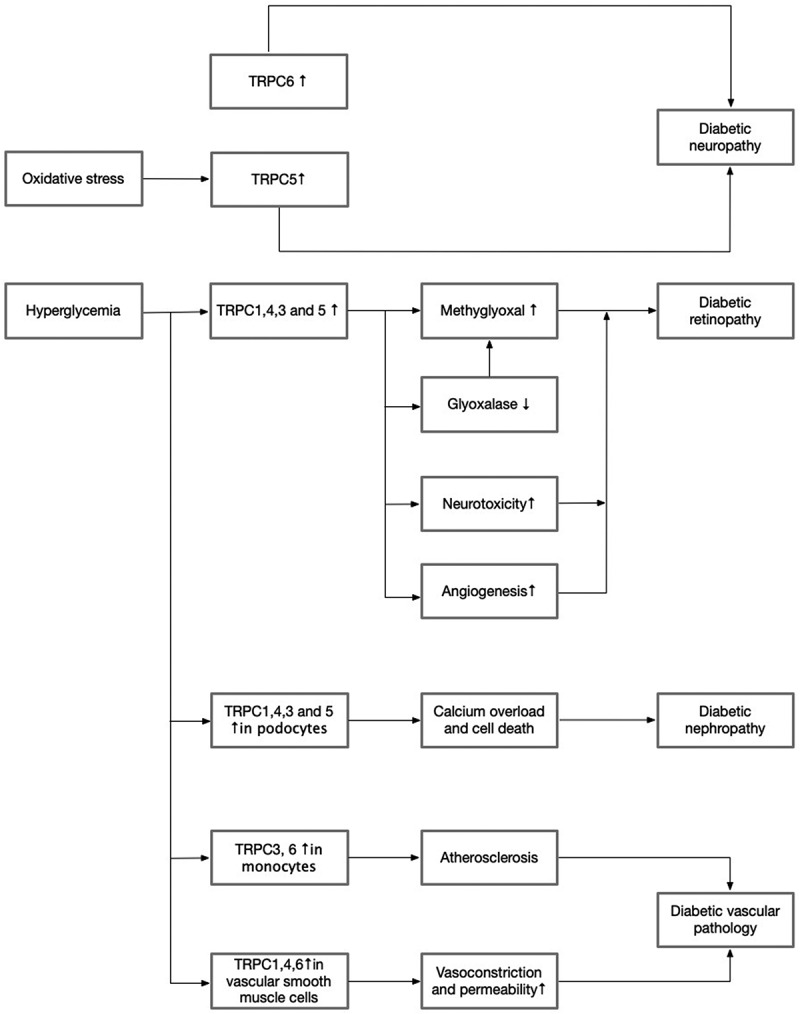
Role of TRPCs in diabetic complications.

T2DM leads to both microvascular complications – such as nephropathy, retinopathy, and neuropathy – and macrovascular complications, including accelerated atherosclerosis that predisposes to premature ischemic heart disease, cerebrovascular events, and peripheral vascular disease (Figure 3). These complications are major contributors to morbidity and mortality in diabetes. Chronic hyperglycemia and the diabetic environment have been shown to modulate TRPC channel expression and function across multiple tissues, including nerves, retina, kidneys, and blood vessels, although these effects are subtype- and tissue-specific [10,12,137].

#### Diabetic neuropathy

Diabetic neuropathy is associated with dysregulated intracellular Ca^2 +^ signaling in peripheral neurons [138]. TRPC channels are nonselective cation channels that permit Ca^2 +^ influx into cells. Therefore, upregulation of TRPC channel activity may lead to excessive Ca^2 +^ entry, which could contribute to the development of diabetic neuropathy.

Several studies have elucidated the role of TRPC channels in diabetic neuropathy. TRPC5 contributes to pain associated with diabetic peripheral neuropathy, and its modulation represents a potential therapeutic strategy. Activation of TRPC5 by BTD (N-[3-(adamantan-2-yloxy)propyl]-3-(6-methyl-1,1-dioxo-2 H-1λ6,2,4-benzothiadiazin-3-yl)-propanamide), a potent TRPC5 activator, alleviated mechanical allodynia in diabetic rats via TRPC5–CAMKII – ERK signaling [139]. However, TRPC5 mediates adrenaline released by adrenal chromaffin cells, which counters hypoglycemia induced by insulin [140].

In models of diabetic neuropathic pain, TRPC6 and BDNF are markedly upregulated in the dorsal root ganglia (DRG) and the spinal cord. This upregulation enhances neuronal excitability through activation of TRPC6-mediated calcium influx and is associated with the development of mechanical allodynia. Moreover, pharmacological inhibition of TRPC6 effectively alleviates pain-related behaviors in diabetic neuropathic pain models [141].

#### Diabetic retinopathy

Diabetic retinopathy (DR), a serious diabetes-related complication, is characterized by microaneurysm formation, pericyte loss, thickening of the basement membrane, disruption of the blood – retina barrier, and pathological neovascularization [142,143]. Chronic hyperglycemia damages retinal small blood vessels, and reactive metabolites (RM) accumulated under hyperglycemia, including reactive carbonyl (RCS), oxygen (ROS), and nitrogen (RNS) species, contribute to disease progression [144]. Methylglyoxal (MG) plays a pivotal role by inducing structural and transcriptional modifications and forming advanced glycation end-products (MG-AGEs) [144–146]. TRPC channels are broadly expressed in the retina and implicated in DR pathogenesis. In STZ-treated mice, TRPC1/4/5/6 depletion protected against DR, reducing hyperglycemia evoked vasoregression, lowering MG levels, and increasing activity of Glyoxalase 1 (GLO1), a key MG detoxifying enzyme [144]. These results indicate that mice lacking TRPC1, TRPC4, TRPC5, or TRPC6 exhibit increased resistance to diabetic retinopathy. In human retinal endothelial cells (HRECs), high glucose upregulates TRPC1 and TRPC6. Inhibition of the TRPC by nonspecific TRPCs channel blocker SKF96365 not only decrease vascular endothelial growth factor (VEGF) expression level, which is an important factor in promoting neovascularization of DR retina and thus has a significant effect in the treatment of DR in the proliferative phase, but also prevent proliferation, migration, and lumen formation of HRECs induced by high glucose [147]. Another study reported that activation of TRPC6 with Hyp9, a highly selective TRPC6 agonist, exacerbated the progression of DR by promoting ROS accumulation and upregulating IL-6 and VEGF expression in cells exposed to high glucose. In contrast, TRPC6 silencing or knockdown attenuated Müller cell dysfunction, preserving cell viability and reducing gliosis in rat Müller cell line (rMC-1) cells, while decreasing IL-6, VEGF, ROS, and intracellular Ca^2+^ levels [148].

#### Diabetic nephropathy

Diabetic nephropathy (DN) is a progressive complication affecting approximately 40% of diabetic patients, leading to a decline in glomerular filtration rate and representing a major cause of end-stage renal disease [149,150]. Early DN is characterized by glomerular hyperfiltration and mesangial extracellular matrix accumulation [151,152], progressing to glomerulosclerosis, renal fibrosis, and renal insufficiency [151,153]. Mesangial cells (MCs) are specialized contractile cells that provide structural support and physiologically regulate glomerular hemodynamics. Weakened MCs contractions contribute to early diabetic hyperfiltration [154,155].

TRPC channels, particularly TRPC6, contribute to diabetic kidney disease (DKD) and high glucose – induced renal injury [156]. Recent research showed that in T2DM mice, TPRC6 knockout mitigated weight loss, reduced fasting blood glucose, and improved renal dysfunction and glomerular fibrosis. Knockout also lowered p-SMAD2/3, TGF‑β, calcineurin (CN), NFAT2, and NLRP3 inflammasome proteins, reduced intracellular Ca^2 +^ ([Ca^2 +^]i), and restored calcium homeostasis, suggesting that TRPC6 deletion alleviates glomerular fibrosis by inhibiting [Ca^2 +^]i overload and the CN – NFAT2 pathway [157]. In diabetes, mesangial contractile hypocontractility is linked to impaired Ca^2 +^ influx; high glucose (30 mM) reduces Angiotensin II – stimulated Ca^2 +^ entry in human MCs via selective TRPC6 downregulation, while TRPC1 and TRPC3 remain unaffected [158]. The NADPH oxidase 4 (Nox4)/ROS/protein kinase C (PKC)/NF-κB signaling pathway is involved in the depletion of TRPC6 [159,160]. However, in contrast to the studies discussed above, Li et al. [161] reported a ~ 3-fold increase in TRPC6 in rat kidneys 12 weeks post-STZ, and high glucose (833 mM, 48 h) increased TRPC6 in cultured rat MCs. This discrepancy may be attributed to differences in the cultured mesangial cell species and the glucose concentrations used.

Plenty of evidence focused on the TRPC6 effect on diabetic podocyte injury. Diabetic podocyte injury is mediated, at least in part, by up-regulation of TRPC6. Diabetes upregulates TRPC6 in podocytes, decreasing autophagic flux and calpastatin expression, which correlates with podocyte injury in patient kidney biopsies [162]. Conversely, metformin reduces TRPC6 levels via AMPK activation, suggesting therapeutic potential for TRPC6 modulation in diabetic complications [10,163,164]. TRPC6 upregulation may be mediated by canonical Wnt signaling, as shown in mouse podocytes exposed to high glucose [165]. Actually, the protective effect after TRPC 6 deletion is reported. In STZ-treated Dahl Salt-sensitive rats, deletion of TRPC6 protected podocytes from injury and H_2_O_2_-induced oxidative damage through NOX4, without altering overall glomerular structure, highlighting TRPC6 and NOX4 as potential therapeutic targets for slowing the progression of diabetic kidney disease [166,167].

Meanwhile, TRPC6 is the major target in kidney disease treatment. The TRPC6 inhibitor BI764798, developed by Boehringer Ingelheim, is currently in Phase 2 clinical trials for Focal Segmental Glomerulosclerosis (FSGS). BI 764,198 lowered proteinuria and was well tolerated by participants in this trial. This is the first evidence of efficacy with a podocyte-targeted therapy in FSGS. BI 764198 was well tolerated with no meaningful differences in adverse event frequencies across treatments [168]. Currently, BI764198 is moving into phase 3 trials for proteinuric kidney diseases. Another oral TRPC6 inhibitor, BI749327, has shown promising progress in animal models for reducing renal fibrosis [169].

In addition, TRPC1 and TRPC3 contribute to diabetic nephropathy. TRPC1, located on human chromosome 3q22-24, a region which is recognized as a susceptibility hotspot for diabetic nephropathy [170], is down-regulated in the kidneys of several type 2 diabetes models, including Zucker fatty rats, STZ-injected rats, and db/db mice, as well as in patients with nodular glomerulosclerosis [170,171]. However, TRPC1 polymorphisms have not been associated with diabetic nephropathy or T2DM-related end-stage renal disease [171], and it remains unclear whether TRPC1 mRNA levels and protein expression are specifically reduced in glomerular mesangial cells. He et al. reported that miR-135a accumulation in serum and renal tissue from DN patients and db/db mice paralleled microalbuminuria and renal fibrosis, and that TRPC1 inhibition facilitated miR-135a – associated mesangial cell pathology [172]. Therefore, unlike TRPC6, TRPC1 appears to suppress mesangial cell proliferation and mitigate glomerular injury in DN. Thrombin, a serine protease that cleaves fibrinogen into fibrin, is a potent profibrotic factor and key contributor to DN [173]. It promotes mesangial remodeling in diabetic patients via protease-activated receptors (PARs) expressed in the renal mesangium. Thrombin also induces synchronous intracellular Ca^2 +^ oscillations in mesangial cells, which depend on PAR1 G-protein – coupled receptor activation and are mediated, at least in part, by SOCE and TRPC3 channel activity [174].

#### Diabetes induced vascular pathology

Diabetes is strongly associated with nephropathy, retinopathy, and neuropathy, all of which increase the risk of cardiovascular disease. Endothelial cells, vascular smooth muscle cells (VSMCs), and platelet dysfunction collectively drive diabetic vascular complications. TRPC channels have emerged as important mediators of these vascular alterations in diabetes and obesity, making them potential targets for preventing or treating related end-organ damage. In vascular tissues and diabetic or obese animal models, the expression of TRPC1, TRPC4, and TRPC6 is altered. For example, in human saphenous veins from T2DM patients, TRPC1 and TRPC6 protein levels were downregulated, whereas TRPC4 mRNA was upregulated. Circulating monocyte recruitment to the arterial intima is a key step in atherosclerosis, and high glucose has been shown to upregulate TRPC3 and TRPC6 proteins as well as TRPC1 and TRPC5 mRNA in human monocytes. Notably, TRPC6 mRNA, but not TRPC1, TRPC3, or TRPC5, was elevated in monocytes from type 2 diabetes patients versus controls [175]. TRPC6 expression is also increased in myocardial fibrosis during diabetic cardiomyopathy, enhancing TGF-β/Smad3 signaling and promoting collagen I (COL-1) synthesis. Treatment with Salvianolic acid B (Sal B), the main water-soluble polyphenolic component of Danshen, inhibited abnormal TRPC6 expression and TGF-β/Smad3 activation, mitigating these effects [176]. Together, these findings suggest that TRPC channels may contribute to the vascular dysfunction observed in diabetes and obesity [137,177].

Expression of TRPC1, TRPC3, TRPC4, TRPC5, and TRPC6 has been identified in VSMCs [178–181] and endothelial cells [182–184], highlighting the importance of investigating their roles in diabetic vascular dysfunction. In cultured aortic smooth muscle cells from diabetic Goto-Kakizaki (GK) rats, Evans et al. reported that TRPC4 was upregulated and TRPC6 downregulated, while TRPC1 and TRPC5 remained unchanged. Angiotensin II (Ang II)-induced Ca^2 +^ influx was enhanced, insensitive to voltage-gated Ca^2 +^ channel inhibition but suppressed by 1-oleoyl-2-acetyl-sn-glycerol (OAG), an activator of TRPC3/6/7 and inhibitor of TRPC4/5, indicating that overactive TRPC1/4/5 channels underlie the elevated Ca^2 +^ entry [178].

SOCE is active in smooth muscle cells and plays a role in mediating vasoconstriction [185]. In diabetes, signaling pathways in vascular tissue are perturbed, resulting in vasomotor dysfunction characterized by impaired responses to vasodilators and exaggerated responses to vasoconstrictors, which can lead to hypertension [186]. Vessels from diabetic patients exhibit increased contractility compared to those from non-diabetic individuals [177], a phenomenon partly attributed to Ca^2 +^ entry through SOCs and alterations in TRPC1, TRPC4, and TRPC6 expression. In the caudal artery smooth muscle of diabetic GK rats, TRPC1 and TRPC6 expression was increased, and TRPC4 was detected, whereas TRPC4 was absent in non-diabetic Wistar rats. These expression changes were associated with reduced cirazoline- or cyclopiazonic acid (CPA)-induced contractions in GK arteries [180]. In human saphenous veins, CPA-induced contractions were more pronounced in diabetic vessels compared to non-diabetic controls, indicating that increased Ca^2 +^ entry via SOC contributes to the heightened vascular contractility observed in diabetes. Notably, TRPC1 and TRPC6 protein levels were decreased in diabetic saphenous veins, while mRNA levels remained unchanged, and TRPC4 mRNA was elevated without corresponding protein changes. Although TRPC protein expression is reduced, the augmented CPA-induced contractions observed in diabetic veins may reflect increased TRPC channel activity, resulting in elevated capacitative Ca^2 +^ entry [177].

Endothelial dysfunction is another key contributor to the abnormal vascular contractility observed in diabetes [187]. In vascular endothelial cells, overactivation of TRPC channels has been implicated in this dysfunction, likely by disrupting Ca^2 +^ homeostasis. Impaired endothelial function has been linked to a Ca^2 +^ -dependent reduction in nitric oxide bioavailability [188]. Because TRPC channels act as receptor- and/or store-operated Ca^2 +^ channels in endothelial cells [189], their dysregulation may contribute to diabetes-associated vascular pathology. Bishara et al. [190] showed that in cultured bovine aortic endothelial cells, high glucose selectively increased TRPC1 protein, enhancing agonist-stimulated Ca^2 +^ entry.

Platelet dysfunction drives the prothrombotic state in diabetes, with type 2 diabetic platelets exhibiting heightened adhesiveness and aggregation [191]. This hyperreactivity is closely linked to disrupted intracellular Ca^2 +^ homeostasis, a key factor in the cardiovascular complications of diabetes [191,192]. Several studies have reported that Tg, a SERCA Ca^2 +^ -ATPase inhibitor, induces greater Ca^2 +^ entry in platelets from diabetic donors, indicating enhanced SOCE [193]. Elevated expression of TRPC3, Orai1, and STIM1 in diabetic platelets suggests that upregulation of these channels contributes to the increased Ca^2 +^ influx [194]. TRPC6 is also expressed in platelets, and high glucose has been shown to increase its surface expression via a phosphatidylinositol 3-kinase – dependent pathway [195]. Platelets from type 2 diabetic patients display higher TRPC6 levels and enhanced TRPC6-mediated, OAG-induced Ca^2 +^ entry, indicating that TRPC6 May drive platelet hyperreactivity in diabetes [195]. Conversely, a recent study reported attenuated SOCE in diabetic platelets, possibly due to impaired interaction between STIM1 and Orai1/TRPC1/TRPC6, despite an overall increase in agonist- or Tg -induced Ca^2 +^ influx [196]. This study indicates that, in addition to SOCE, store-independent Ca^2 +^ entry pathways may also contribute to the elevated platelet activity observed in type 2 diabetes, although the precise underlying mechanisms remain incompletely understood.

### TPRC channels on obesity

Obesity is strongly linked to the development of type 2 diabetes, and the transition from obesity to diabetes results from progressive impairment of insulin secretion and increasing insulin resistance. This interrelationship, often termed “diabesity,” arises from shared etiological factors such as excessive fat accumulation and associated metabolic and cellular alterations [197]. TRPC1 channels have been shown to counteract the protective effects of exercise against obesity and diabetes in high-fat diet conditions. TRPC1 knockout mice are resistant to diet-induced obesity and insulin resistance when exercised, likely due to reduced adiposity, mediated by decreased adipose autophagy and increased adipose apoptosis. Calcium influx through TRPC1 channels appears to mediate the inhibitory effects of TRPC1 on exercise induced metabolic benefits [121].

Macrophages play a critical role in adipose tissue inflammation and metabolic regulation. Under obese conditions, macrophages tend to polarize toward the pro-inflammatory M1 phenotype, promoting inflammation, lipid accumulation, and systemic insulin resistance. Conversely, polarization toward the anti-inflammatory M2 phenotype is associated with reduced inflammation, improved insulin sensitivity, and increased white adipose tissue browning [198]. Lin Y et al. reported that expression of IL-6 and macrophage-related chemokines (Ccl4, Ccl5, and Cxcl9) was markedly elevated in the adipose tissue of TRPC knockout mice, suggesting an increase in M1-type macrophages. The loss of TRPC also enhanced secretion of pro-inflammatory cytokines, including IL-6, IL-12, and TNF-α, by M1 bone marrow – derived macrophages, suggesting that TRPC channels participate in immune-mediated pathways that contribute to insulin resistance [199]. These findings indicate that TRPC channels modulate adipose inflammation and energy metabolism, linking their dysfunction to the pathogenesis of obesity and diabetes.

## Conclusion

Although TRPC channels have gained increasing attention for their physiological relevance, their specific roles in pancreatic β cells and the pathogenesis of diabetes remain insufficiently characterized. While several studies have begun to explore this area, substantial knowledge gaps persist. Notably, most investigations related to diabetic complications have focused on TRPC6, whereas the contributions of other TRPC isoforms, such as TRPC1, TRPC3, and TRPC4, remain poorly defined. Moreover, much of the current understanding of TRPC channel function in insulin resistance arises from podocyte and renal models, rather than from canonical insulin responsive tissues such as skeletal muscle, liver, or adipose tissue. This tissue bias limits the extrapolation of its translational progress and drug development.

Another major limitation lies in the predominantly preclinical nature of existing studies. Most data are obtained from cell culture or animal models, with limited clinical or translational evidence to confirm the physiological and pathological roles of TRPC channels in humans. Furthermore, certain studies report inconsistent findings. For instance, some evidence suggests that TRPC3 facilitates GSIS in β cells, whereas other studies suggest that TRPC3 inhibition exerts no effects on either basal or stimulated insulin release. Such discrepancies may reflect differences in experimental models, species, or methodological approaches.

Collectively, while the emerging evidence underscores the potential importance of TRPC channels in β cell function and diabetic pathology, their precise molecular mechanisms, tissue-specific roles, and therapeutic potential remain to be fully elucidated. Future research integrating multimodal approaches, including human studies and genetic or pharmacological modulation of TRPC activity, will be essential to clarify their contributions and evaluate their viability as novel therapeutic targets in diabetes and its complications.

## Data Availability

Data sharing is not applicable to this article as no data were created or analyzed in this study.
